# Nationwide characterization of labor neuraxial analgesia provision in Japan using a publicly accessible database

**DOI:** 10.1007/s00540-025-03611-w

**Published:** 2025-10-25

**Authors:** Daisuke Sakamaki, Kazutoshi Onitsuka, Yusuke Mazda

**Affiliations:** 1 Department of Anesthesiology, Jikei University School of Medicine, Tokyo, Japan; 2 Department of Anesthesiology and Critical Care Medicine, Graduate School of Medical and Dental Sciences, Kagoshima University, Kagoshima, Japan; 3 Department of Obstetric Anesthesiology, Center for Maternal-Fetal and Neonatal Medicine, Saitama Medical Center, Saitama Medical University, 1981 Kamoda, Kawagoe, Saitama 350-8550 Japan

**Keywords:** Japan, Labor neuraxial analgesia, National database

## Abstract

**Background:**

In Japan, labor neuraxial analgesia (LNA) is frequently administered by obstetricians rather than board-certified anesthesiologists, particularly in smaller facilities. Although awareness of maternal safety has increased in recent years, the extent of anesthesiologist involvement in obstetric anesthesia remains unclear.

**Methods:**

This nationwide cross-sectional study analyzed data from Birth-Navi, a public registry of childbirth facilities maintained by Ministry of Health, Labour and Welfare of Japan. As of August 2024, facilities offering LNA were categorized into two groups: those listing a board-certified anesthesiologist (Group A) and an obstetrician-gynecologist (Group O) as a responsible physician. Institutional characteristics and analgesia practices were compared between groups using chi-square tests.

**Results:**

Among 2063 registered facilities, 837 (40.6%) provided LNA, of which 771 met the inclusion criteria. Only 27.2% facilities listed a board-certified anesthesiologist as the responsible physician. Group A facilities were more likely to be hospitals (86.8% vs. 30.4%, p < 0.001) and more likely to utilize combined spinal–epidural techniques (23.7% vs. 14.0%, p = 0.002). However, 24 h analgesia availability was significantly lower in Group A than in Group O (25.9% vs. 47.1%, p < 0.05). Notably, only 284 facilities (13.8%) provided round-the-clock analgesia upon maternal request.

**Conclusion:**

It is important to note that anesthesiologist-led LNA remains limited in Japan. While associated with more advanced techniques, 24 h availability is uncommon. To improve both access and safety, system-level strategies—such as redistribution of personnel and the implementation of collaborative tele-anesthesia networks—should be considered.

## Introduction

In Japan, labor neuraxial analgesia (LNA) is frequently administered without the involvement of board-certified anesthesiologists, particularly in small-scale childbirth facilities. Approximately 45.3% of deliveries occur in inpatient clinics with fewer than 20 beds [1], where obstetricians often assume dual responsibilities, managing both labor and anesthesia. This decentralized but functionally coordinated perinatal system contributes to considerable variability in LNA availability and qualifications of attending providers across the country.

Despite increasing emphasis to maternal safety and patient-centered care, the actual extent of anesthesiologist involvement in LNA remains poorly characterized. A key obstacle has been the absence of a centralized, comprehensive database encompassing both provider qualifications and institutional capabilities. In response to growing demand for transparency, the Ministry of Health, Labour and Welfare launched Birth-Navi, a nationwide, publicly accessible registry of childbirth facilities that covers approximately 99% of delivery-capable institutions in Japan [2]. This database provides detailed information on facility type, delivery volume, LNA techniques, and the credentials of responsible physicians.

While LNA is a standard component of perinatal care in many high-income countries, Japan continues to face challenges in achieving widespread and equitable access. In the United States, for example, over 70% of vaginal deliveries involve neuraxial techniques, typically administered by anesthesiologists under standardized protocols. Similar practices are observed in Canada and parts of Europe, where anesthesiologist participation in LNA is routine and embedded within multidisciplinary care models. In contrast, Japan lacked the infrastructure to evaluate national practices until the recent implementation of Birth-Navi.

To our knowledge, the present study is the first to utilize this comprehensive registry for a nationwide assessment of anesthesiologist involvement in LNA. By leveraging the unique feature of Birth-Navi, we aim to provide a granular overview of current clinical practices and highlight structural disparities across facility types in Japan.

## Methods

This cross-sectional study utilized data from Birth-Navi, an online database published by Japan’s Ministry of Health, Labour and Welfare. As of April 2024, eligible facilities were defined by the following criteria: (1) currently offering childbirth services; (2) classified as a hospital, an inpatient clinic with fewer than 20 beds, or a midwifery center; and (3) having performed at least 21 deliveries during fiscal year 2023 and participating in the direct payment system for the lump-sum birth allowance. Additionally, facilities fulfilling criteria (1) and (2) were eligible to voluntarily enroll in the registry regardless of their annual delivery volume.

Birth-Navi contains comprehensive institutional data, including facility type, number of deliveries, midwifery staffing, availability and method of LNA, qualifications of responsible clinicians, service hours and related fees. SInce Birth-Navi covers nearly all major childbirth facilities nationwide, it is regarded as a reliable and accessible resource for residents regardless of geographic location. As this study relied solely on publicly available data, institutional ethical review was not required.

### Study protocol

All childbirth facilities listed in Birth-Navi as of August 15, 2024, were screened for inclusion. Facilities classified as midwifery centers were excluded. We identified hospitals and inpatient clinics that reported the availability of LNA services. Among these, we selected facilities that explicitly identified the provider responsible for LNA as either a board-certified anesthesiologist (Group A) or a board-certified obstetrician-gynecologist (Group O). Facilities were subsequently categorized into these two groups for comparative analysis. We compared institutional characteristics and LNA practices between Group A and Group O.

### Outcomes

The primary outcome was the proportion of childbirth facilities in which LNA was managed by a board-certified anesthesiologist. Secondary outcomes included differences in facility characteristics and analgesic technique preferences between the two groups.

### Statistical analysis

Comparative analyses between Group A and Group O were performed using the chi-square test. A two-tailed p-value of less than 0.05 was considered statistically significant. All statistical analyses were conducted using commercially available statistical software (Microsoft Excel, Microsoft Corp., Redmond, WA, USA).

## Results

**Fig. 1:**
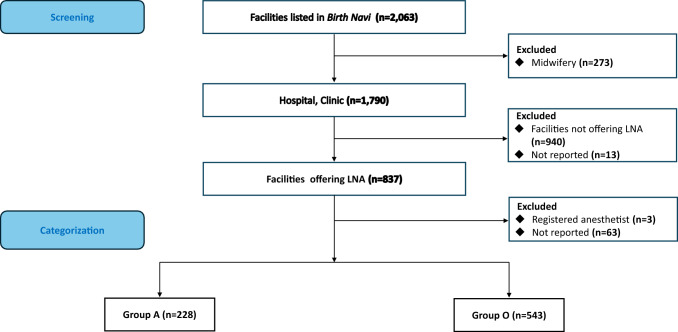
The flow diagram. *LNA* labor neuraxial analgesia. Group A is a facility with a responsible physician for LNA as a board-certified anesthesiologist, and Group O is a facility with a responsible physician for LNA as a board-certified obstetrician-gynecologist. Registered anesthetist is a physician with proper training for anesthesia, but not board-certified

Of the 2063 childbirth facilities registered in Birth-Navi, a total of 273 facilities classified as midwifery centers and 953 facilities that either did not offer LNA (n = 940) or lacked identifiable information on LNA (n = 13). The remaining 837 facilities (40.6% of all registered institutions) reported providing some form of LNA. An additional 66 facilities were excluded due to missing provider information or the absence of relevant board certification. As a result, 771 facilities were included in the final analysis. Of these, 228 were classified into Group A, and 543 into Group O (Fig. 1).

Although 652 facilities (31.6%) reported offering LNA upon maternal request, only 284 (13.8%) provided this service around the clock. By contrast, 119 facilities limited analgesia provision strictly to cases with medical indications. Many of these institutions appeared to lack the structural capacity to provide timely responses, raising concerns about the consistency and reliability of service delivery in such settings.

Across all facilities offering LNA, only 27.2% listed a board-certified anesthesiologist as the responsible clinician. Group A facilities were significantly more likely to be hospitals compared to Group O (86.8% vs. 30.4%, p < 0.001; Table 1). Additionally, the use of combined spinal–epidural analgesia (CSEA) was more frequent in Group A (23.7% vs. 14.0%, p = 0.002).

**Table 1:** Facility characteristics by responsible physician type for LNA

	Total n = 771	Group A n = 228	Group O n = 543	p value
Facility type*, n (%)*				< 0.001
Hospital (beds ≥ 20)	363	198 (86.8)	165 (30.4)	
Clinic (beds < 20)	407	30 (13.2)	378 (69.6)	
Indication for LNA*, n (%)*				< 0.001
Maternal request	652	178 (78.1)	474 (87.3)	
Medical indication only	119	50 (21.9)	69 (12.7)	
Type of LNA, n (%)*				
Epidural	744	218 (95.6)	526 (96.9)	0.515
CSEA	130	54 (23.7)	76 (14.0)	< 0.002
Others	41	14 (6.1)	27 (5.0)	0.629
Availability of 24 h service,* n (%)*			< 0.001	
Yes	315	59 (25.9)	256 (47.1)	
No	420	159 (69.7)	261 (48.1)	
Not reported	36	10 (4.4)	26 (4.8)	
LNA availability by onset of labor*, n (%)*			< 0.001	
Both spontaneous and induced labor	222	35 (15.4)	187 (34.4)	
Induced labor only	496	181 (79.4)	315 (58.0)	
Not reported	53	12 (5.3)	41 (7.6)	
Listed on the JALA registry*, n (%)*				0.961
Yes	346	103 (45.2)	243 (44.8)	
No	409	120 (52.6)	289 (53.2)	
Not reported	16	5 (2.2)	11 (2.0)	

Clinic is defined as a medical facility with fewer than 20 inpatient beds. *Multiple responses were allowed

*LNA* labor neuraxial analgesia, *CSEA* combined spinal–epidural analgesia, *JALA* Japanese Association for Labor Analgesia

It is noteworthy that the rate of registration with Japanese Association for Labor Analgesia (JALA)—a collaborative organization aimed at enhancing the safety of obstetric anesthesia—was nearly identical between the two groups (45.2% vs. 44.8%, p = 0.961).

## Discussion

This study revealed that 40.6% (837 facilities) of childbirth centers listed in the Birth-Navi database offered LNA, and among them, 31.6% (652 facilities) provided analgesia upon maternal request. However, only 13.8% (284 facilities) could offer LNA on a 24 h basis. Moreover, only 27.2% of these analgesia-providing facilities had a board-certified anesthesiologist designated as the person in charge. Compared with smaller facilities, Group A facilities were significantly more likely to be hospitals (86.8% vs. 30.4%, p < 0.05).

International comparisons highlight the unique circumstances in Japan. Although the use of labor neuraxial analgesia has been gradually increasing in Japan, the implementation rate remains as low as 13.8% [3]. In contrast, over 70% of deliveries in the United States involve labor neuraxial analgesia, nearly all administered under the supervision of anesthesiologists [4]. In Canada, the rate varies by province but has been reported at 59% [5]. In these countries, anesthesiologists play a central role in labor pain management, supported by institutional protocols and multidisciplinary care systems.

In Japan, obstetricians were classically responsible for administering anesthesia, reflecting the country’s reliance on small-scale obstetric clinics, which account for 45.3% of all deliveries [1], as well as historical shortage of anesthesiologists in the labor floor. This study similarly showed that Group O facilities were more likely to be clinics (13.2% vs. 69.6%, p < 0.05) and had a higher rate of 24-h analgesia availability (25.9% vs. 47.1%, p < 0.05). These facilities tend to operate under a decentralized care model led by obstetricians. While this model is suitable for low-risk deliveries, a structured referral system ensures that high-risk cases are transferred to advanced medical centers, resulting in a functionally coordinated perinatal care network.

The involvement of anesthesiologists has been associated with improved clinical outcomes. Previous studies have shown that LNA administered by anesthesiologists is linked to higher maternal satisfaction, more effective pain relief, and reduced rates of conversion to general anesthesia during cesarean sections [6]. Additionally, guidelines from the American Society of Anesthesiologists and the American College of Obstetricians and Gynecologists emphasize the importance of standardized analgesia protocols, drug management, and complication response systems to enhance both safety and consistency in care [7]. In the present study, the use of CSEA was significantly more frequent in Group A than in Group O (23.7% vs. 14.0%, p = 0.002), suggesting that facilities managed by anesthesiologists are more capable of offering diverse analgesic options.

The establishment of JALA in 2017 marked a turning point in the promotion of interdisciplinary collaboration and the development of safer LNA systems in Japan [8]. Although JALA has proposed a framework for facility standards, its implementation has been inconsistent. Our previous nationwide survey found that only 32% of JALA-registered facilities offering labor neuraxial analgesia had full-time anesthesiologists [9]. However, the study did not assess the actual involvement of anesthesiologists in clinical practice. In contrast, the current study focused specifically on whether an anesthesiologist was designated as the responsible provider, offering a more accurate reflection of clinical reality.

To expand anesthesiologist involvement in perinatal care, practical and sustainable strategies are urgently needed. Although the number of board-certified anesthesiologists in Japan has increased by approximately 14% over the past decade [10, 11], their responsibilities extend beyond surgical anesthesia to include non-operating room anesthesia, critical care, pain management, and palliative care. Therefore, dedicating anesthesiologists exclusively to LNA is often impractical, especially for 24 h service that includes spontaneous labor and overnight coverage. Under these circumstances, feasible steps might include implementing models such as rotating anesthesiologists limited to scheduled or induced labors or providing telemedicine support specialized for preoperative assessment and non-urgent interactions (such as anesthetic explanation or consultation). In the United States, team-based care models involving anesthesiologists, obstetricians, and midwives have contributed significantly to improving safety in perinatal medicine [12]. Furthermore, the use of non-physician providers like Certified Registered Nurse Anesthetists is institutionally accepted in the United States, offering a potentially valuable perspective for discussions on system design in Japan. However, the use of non-physician providers remains legally prohibited in Japan, and its feasibility and safety must be carefully evaluated. Nevertheless, these alternative models for regional anesthesiologist deployment or telemedicine support must be understood to entail numerous operational challenges. In particular, approximately 45.3% of deliveries in Japan occur at small obstetric clinics, many of which lack sufficient delivery volume or medical resources to support full-time anesthesiologists. Moreover, in emergent events such as massive hemorrhage or high spinal anesthesia, immediate direct intervention is required; telemedicine alone is likely insufficient. Several recent studies have highlighted limitations of the current decentralized obstetric anesthesia system and emphasized the need for consolidating deliveries in better-equipped large hospitals to improve maternal and fetal outcomes [13–15].

In addition, while providing LNA itself does not necessarily require board certification in anesthesiology, the *responsible physician* should be a board-certified anesthesiologist. In many countries, LNA is performed by anesthesia residents under supervision of board-certified anesthesiologists. Similarly, in Japan, physicians who have acquired sufficient skills through formal anesthesiology training can safely perform the neuraxial procedure itself. However, the ultimate accountability for safety and quality must rest with anesthesiologists. This responsibility should not depend on an individual’s preference or willingness to specialize in obstetric anesthesia but should instead be recognized as an integral professional duty—just as general surgeons are expected to perform appendectomies as part of their scope of practice. Clarifying this distinction between “provider” and “responsible physician” may help establish a realistic and sustainable framework for anesthesiologist involvement in LNA. While regional and logistical barriers exist, a long-term strategy to correct structural inequalities in obstetric anesthesia care remains warranted.

### Limitations

This study has several limitations. First, while previous studies have focused on the presence of full-time board-certified anesthesiologists, we examined facilities where anesthesiologists were officially registered as the provider responsible for LNA. These differing definitions limit direct comparability. Second, the study did not capture who performed the analgesia procedures at each facility. For example, although an obstetrician may have been listed as the responsible provider, the procedures might have been carried out by an anesthesiologist, making the reported 27.2% potentially a lower bound for anesthesiologist involvement. Third, because Birth-Navi is a relatively new database, some registered information may no longer reflect the current staffing situation due to personnel changes after registration. Fourth, information regarding the availability of 24 h LNA was based on self-reported data submitted by each facility to the Birth-Navi registry. As such, there may be discrepancies between the reported availability and the actual capacity for around-the-clock service, particularly in facilities with limited staffing or infrastructure.

### Implications and future directions

This study is the first nationwide analysis to evaluate LNA systems in Japan using the comprehensive Birth-Navi database. It found that only about one-quarter of facilities offering analgesia had a board-certified anesthesiologist registered as the responsible provider. These facilities were more likely to be hospitals with better staffing and infrastructure than small clinics, yet many still lacked systems capable of responding promptly to spontaneous or nighttime labor.

To establish a sustainable 24 h LNA system, it is essential to create a clinical environment where anesthesiologists can be involved safely and efficiently. However, assigning full-time anesthesiologists to every facility is unrealistic. Thus, resource-conscious and pragmatic strategies must be developed. Strengthening collaboration between anesthesiologists and obstetricians will be crucial. Standardized guidelines for neuraxial block techniques, unified protocols for drug management and complication response, and the implementation of interdisciplinary safety frameworks are likely to be key to improving both the quality and accessibility of LNA services across Japan. Future research should investigate how anesthesiologists balance LNA responsibilities with surgical anesthesia workloads and evaluate the feasibility of structured collaboration models that include task-sharing protocols and scheduled coverage for LNA.

## Glossary

CSEA: Combined spinal–epidural analgesia.
JALA: Japanese Association for Labor Analgesia.
LNA: Labor neuraxial analgesia.

## References

[1] Ministry of Health, Labour and Welfare of Japan. 2020 Summary of Static/Dynamic Surveys of Medical Institutions and Hospital Report. Available from: https://www.mhlw.go.jp/english/database/db-hss/mih_report_2020.html. Accessed Oct 11, 2025.

[2] Ministry of Health, Labour and Welfare of Japan. *Birth-navi*. Available from: https://www.mhlw.go.jp/stf/birth-navi/index.html. Accessed Oct 11, 2025.

[3] Ministry of Health, Labour and Welfare of Japan. 2023 Summary of Static/Dynamic Surveys of Medical Institutions and Hospital Report. Available from: https://www.mhlw.go.jp/english/database/db-hss/mih_report_2023.html. Accessed Oct 11, 2025.

[4] Butwick AJ, Bentley J, Wong CA, Snowden JM, Sun E, Guo N. United States state-level variation in the use of neuraxial analgesia during labor for pregnant women. JAMA Netw Open. 2018;1(8):e186567. 10.1001/jamanetworkopen.2018.6567. 30646335 10.1001/jamanetworkopen.2018.6567 PMC6324365

[5] Canadian Institute for Health Information. Inpatient Hospitalization, Surgery, Newborn, Alternate Level of Care and Childbirth Statistics, 2017–2018. Available from: https://www.cihi.ca/sites/default/files/document/dad-hmdb-childbirth-quick-stats-2017-2018-snapshot-en-web.pdf. Accessed Oct 11, 2025.

[6] Callahan EC, Lee W, Aleshi P, George RB. Modern labor epidural analgesia: implications for labor outcomes and maternal-fetal health. Am J Obstet Gynecol. 2023;228(5S):S1260–9. 10.1016/j.ajog.2022.06.017. 37164496 10.1016/j.ajog.2022.06.017

[7] Rooke DA, Kertai MD, Abrams B. Advancing the role of the anesthesiologist in perioperative medicine. Semin Cardiothorac Vasc Anesth. 2023;27(4):249–51. 10.1177/10892532231212593. 37909211 10.1177/10892532231212593

[8] The Japanese Association for Labor Analgesia. Recommendations for the Safe Provision of Labor Analgesia (in Japanese). 2018. Available from: https://www.jalasite.org/doc/. Accessed Oct 11, 2025.

[9] Mazda Y, Uokawa R, Tanabe S, Ootaki C. Current situation of labor epidural analgesia in Japan: a cross-sectional study. Int J Obstet Anesth. 2020;44:56–7. 10.1016/j.ijoa.2020.07.001. 32799067 10.1016/j.ijoa.2020.07.001

[10] Japan Physicians Association, Japan Specialist Physician System Overview 2022 Edition. Available from: https://jmsb.or.jp/wp-content/uploads/2023/04/gaiho_2022.pdf. Accessed Oct 11, 2025.

[11] Komori M, Nishiyama K, Ichikawa J, Kodaka M, Tomizawa Y. Current problems and working status of female anesthesiologists in Japan. Surg Today. 2014;44(5):982–4. 10.1007/s00595-013-0670-x. 23884564 10.1007/s00595-013-0670-x

[12] Rollins M, Lucero J. Overview of anesthetic considerations for Cesarean delivery. Br Med Bull. 2012;101:105–25. 10.1093/bmb/ldr050. 22219238 10.1093/bmb/ldr050

[13] Maeda A, Mazda Y, Ohara R, Tanabe S, Tokiwa M, Camann W. Obstetric anesthesia in Japan: an existential crisis in need of an intervention. Int J Obstet Anesth. 2025;63:104690. 10.1016/j.ijoa.2025.104690. 40505294 10.1016/j.ijoa.2025.104690

[14] Hasegawa J, Kato R, Terui K, Tanaka H, Tanaka K, Okutomi T, et al. Maternal mortality associated with anaesthetic interventions: nationwide analysis of maternal mortality in Japan from 2010 to 2022. Br J Anaesth. 2025;134(5):1537–40. 10.1016/j.bja.2024.12.011. 39837696 10.1016/j.bja.2024.12.011

[15] Ohara R. Enhancing perinatal safety with the advancement of obstetric anesthesia in Japan. Womens Health Rep (New Rochelle). 2025;6(1):60–8. 10.1089/whr.2024.0154. 39882143 10.1089/whr.2024.0154 PMC11773171

